# Cytoreduction and HIPEC in the treatment of “unconventional” secondary peritoneal carcinomatosis

**DOI:** 10.1186/s12957-015-0703-6

**Published:** 2015-10-22

**Authors:** Maurizio Cardi, Paolo Sammartino, Valentina Mingarelli, Simone Sibio, Fabio Accarpio, Daniele Biacchi, Daniela Musio, Bianca Sollazzo, Angelo Di Giorgio

**Affiliations:** UOC Tecnologie Chirurgiche e Day Surgery, Dipartimento di Chirurgia “P. Valdoni”, “Sapienza” Università di Roma, Rome, Italy; Dipartimento di Scienze Radioterapiche, Oncologiche ed Anatomopatologiche, “Sapienza” Università di Roma, Rome, Italy; Via Bolzano 32, 00198 Rome, Italy

**Keywords:** HIPEC, Cytoreduction, Peritoneal metastasis, Peritonectomy procedures

## Abstract

**Background:**

Peritoneal metastasis (PM) is considered a terminal and incurable disease. In the last 30 years, cytoreductive surgery (CRS) and hyperthermic intraperitoneal chemotherapy (HIPEC) radically changed the therapeutic approach for these patients and is regarded as the standard of care for pseudomyxoma peritonei from appendiceal cancer and peritoneal mesotheliomas. Improved survival has also been reported in treating PM from ovarian, gastric, and colorectal cancers.

However, PM often seriously complicates the clinical course of patients with other primary digestive and non-digestive cancers. There is increasing literature evidence that helped to identify not only the primary tumors for which CRS and HIPEC showed a survival advantage but also the patients who may benefit form this treatment modality for the potential lethal complications.

Our goal is to report our experience with cytoreduction and HIPEC in patients with PM from rare or unusual primary tumors, discussing possible “unconventional” indications, outcome, and the peculiar issues related to each tumor.

**Methods:**

From a series of 253 consecutive patients with a diagnosis of peritoneal carcinomatosis and treated by CRS and HIPEC, we selected only those with secondary peritoneal carcinomatosis from rare or unusual primary tumors, excluding pseudomyxoma peritonei, peritoneal mesotheliomas, ovarian, gastric, and colorectal cancers. Complications and adverse effects were graded from 0 to 5 according to the WHO Common Toxicity Criteria for Adverse Events (CTCAE). Survival was expressed as mean and median.

**Results:**

We admitted and treated by CRS and HIPEC 28 patients with secondary peritoneal carcinomatosis from rare or unusual primary tumors. Morbidity and mortality rates were in line with those reported for similar procedures. Median survival for the study group was 56 months, and 5-year overall survival reached 40.3 %, with a difference between patients with no (CC0) and minimal (CC1) residual disease (52.3 vs. 25.7), not reaching statistical significance. Ten patients are alive disease-free, and eight are alive with disease.

**Conclusions:**

Cytoreduction and HIPEC should not be excluded “a priori” for the treatment of peritoneal metastases from unconventional primary tumors. This combined therapeutic approach, performed in an experienced center, is safe and can provide a survival benefit over conventional palliative treatments.

## Background

Patients with peritoneal metastasis (PM) are typically considered as having a terminal and incurable disease and justifiably treated only by palliation with a very poor prognosis [1, 2]. Although ovarian cancer is one of the most chemotherapy-sensitive solid tumors and one of the few for which the 5-year survival rate has improved, long-term survival in most women with locally advanced disease remains well below 20 % [3–5]. Survival for PM from non-gynecologic malignancies is even worse. The EVOCAPE 1 multicenter study reports a median survival in patients treated with standard surgical and/or chemotherapy regimens of 6.5 and 5.2 months, respectively, in patients with primary gastric and colorectal cancer [6].

Over the past two decades, a novel therapeutic approach has emerged, combining cytoreductive surgery (CRS), performed to treat all visible disease, and hyperthermic intraperitoneal chemotherapy (HIPEC) used to treat microscopic residual disease [7, 8]. This treatment radically changed the therapeutic approach for patients with peritoneal surface malignancies and is nowadays regarded as the standard of care for pseudomyxoma peritonei from appendiceal cancer and peritoneal mesotheliomas [9, 10]. In the last two decades, many studies also reported with this combined approach improved survival for the treatment of peritoneal metastases from ovarian [11–13], gastric [14, 15], and colorectal cancers [16–18].

Peritoneal metastases often complicate also the clinical course of many patients with other primary digestive and non-digestive cancers [19, 20]. Due to the rarity of these conditions, the design of randomized clinical trials of CRS and HIPEC in these patients is unlikely. However, PM is frequently long-term confined to the peritoneal cavity without distant metastases, and death typically occurs for intractable bowel obstruction, development of malignant ascites and mesentery retraction, that often make it impossible to perform even the limited palliative surgery like a simple ostomy. A regional approach seems therefore reasonable in selected patients. Some medical oncologists remain skeptical mostly because of the complexity of the treatment and the perceived high complication rate [21] and the need to treat the patients only in highly specialized centers [22], but despite skepticism, many are the reports of CRS and HIPEC in the treatment of PM in these patients. Expanding literature reports helped to identify not only the primary tumors for which CRS/HIPEC offers a clear survival advantage but also the patients with rare or unusual primary (“unconventional”) cancers who may benefit from this treatment modality for the potential lethal complications and survival advantage [23–25].

Our goal is to report our single-institution experience with CRS and HIPEC in patients with PM from rare or unusual primary tumors, discussing possible indications, outcomes, and the peculiar issues related to each tumor, hoping to contribute to extend the actual knowledge on the treatment of PM by this combined treatment.

## Methods

From the clinical records of a series of 253 consecutive patients admitted in our Institution from November 2000 to December 2013 with a diagnosis of peritoneal carcinomatosis from various primary tumors and treated by maximal cytoreduction and HIPEC, we considered for this study only the patients with a diagnosis of secondary peritoneal carcinomatosis from “unconventional” primary tumors. All patients with primary peritoneal carcinomatosis and with secondary peritoneal carcinomatosis from ovarian, gastric, colorectal, and peritoneal mucinous adenocarcinoma of the appendix (PMCA) were excluded.

All patients gave informed written consent and had a clear histologic diagnosis of peritoneal carcinomatosis. We included only patients with a performance status of 0–2 [26], adequate cardiac, renal, pulmonary and bone marrow function, and resectable disease. Exclusion criteria were extraperitoneal spread, other malignancies, unresectable disease, and severe associated medical conditions.

At laparotomy extent of peritoneal carcinomatosis (PC) was recorded using the peritoneal cancer index (PCI) [27]. Complete surgical cytoreduction was then carried out to resect all visible disease.

Completeness of cytoreduction (CC) was recorded as proposed by Sugarbaker [28].

At the end of the surgical procedure, HIPEC was given with the closed technique. Four drains were positioned and connected to a closed extraperitoneal sterile circuit in which 4 to 6 L of perfusate was circulated by a peristaltic pump at a flow rate of 500 mL/min. The circuit was heated using an external heat exchanger connected to a heating circuit (EXIPER, Euromedical Italy). HIPEC was given at a temperature of 42–43 °C for 60 min using various chemotherapeutic drugs according to the primary tumor (Table 1). At the end, the abdomen was washed with 3–4 L of sterile saline solution at 37 °C.

**Table 1:** Patients clinical characteristics and survival

PT	Age	Sex	Primary T	HIPEC	PCI	CC	FU	Surv
1	72	M	Sarcoma	OXAL^a^	20	1	DOD^c^	12
2	77	F	Sarcoma	OXAL^a^	16	0	AWD^d^	11
3	61	M	Sarcoma	OXAL^a^	14	1	AWD^d^	9
4	68	F	Small bowel	CDDP^b^	26	0	ADF^e^	23
5	51	M	Small bowel	CDDP^b^	15	0	AWD^d^	23
6	59	M	Small bowel	CDDP^b^	20	1	AWD^d^	8
7	46	F	Small bowel	CDDP^b^	7	0	AWD^d^	3
8	67	M	Pancreas	OXAL^c^	23	1	ADF^e^	5
9	67	M	Pancreas	OXAL^c^	22	2	AWD^d^	4
10	74	F	Pancreas	OXAL^c^	3	0	ADF^e^	8
11	70	F	GIST^f^	CDDP^b^	6	0	ADF^e^	34
12	53	F	GIST^f^	CDDP^b^	12	0	ADF^e^	108
13	73	M	GIST^f^	CDDP^b^	20	0	DOD^c^	38
14	58	F	Breast IDC^g^	CDDP^b^	15	0	ADF^e^	128
15	54	F	Breast ILC^h^	CDDP^b^	22	1	ADF^e^	74
16	55	F	Breast ILC^h^	CDDP^b^	22	2	DOD^c^	56
17	77	F	Breast IDC^g^	CDDP^b^	24	1	ADF^e^	45
18	53	F	Breast IDC^g^	CDDP^b^	18	0	ADF^e^	13
19	60	M	Bladder	CDDP^b^	19	2	DOD^c^	9
20	68	F	Type II UPSC^i^	CDDP^b^	5	0	DOD^c^	46
21	56	F	Type II UPSC^i^	CDDP^b^	6	0	DOD^c^	24
22	64	F	Type II UPSC^i^	CDDP^b^	23	0	AWD^d^	12
23	58	F	Type II UPSC^i^	CDDP^b^	17	0	AWD^d^	52
24	61	F	Type II UPSC^i^	CDDP^b^	9	0	ADF^e^	95
25	67	F	Type II UPSC^i^	CDDP^b^	30	1	DOD^c^	15
26	59	F	Type II UPSC^i^	CDDP^b^	29	1	DOD^c^	15
27	65	F	Type II UPSC^i^	CDDP^b^	19	0	DOD^c^	12
28	51	M	Lung	CDDP^b^	19	0	DOD^c^	7

^a^Oxaliplatin

^b^Cisplatin

^c^Died of disease

^d^Alive with disease

^e^Alive disease-free

^f^Gastrointestinal stromal tumor

^g^Invasive ductal carcinoma

^h^Invasive lobular carcinoma

^i^Type II uterine papillary serous carcinoma

Surgical complications and adverse effects were monitored and graded from 0 to 5 (0—No event; 1—Mild; 2—Moderate; 3—Severe; 4—Life-threatening; 5—Death-related) according to the World Health Organization Common Toxicity Criteria for Adverse Events (CTCAE) [29].

The medical oncologic staff planned systemic chemotherapy when deemed necessary. Patients were followed up every 3 months with clinical evaluation and serum-marker monitoring. Imaging techniques were obtained if indicated by the patient’s clinical presentation.

Survival was expressed as mean and median. The Kaplan-Meier method was used to construct survival curves, and log-rank test was used to assess the significance of the differences (cutoff values *p* < 0.05).

## Results

A total of 28 patients with secondary peritoneal carcinomatosis from unconventional primary tumors were admitted and treated by CRS and HIPEC in our Institution. The clinical characteristics and type of primary tumor are reported in Table 1. Mean PCI was 17.1. Twenty-five patients (89 %) had an optimal cytoreduction (17 CC0 and 8 CC1) while three (10.7 %) had CC2 residual disease. Peritonectomy procedures lasted a mean of 475 min (range 300–780) including 60 min of HIPEC. All operations led to major blood loss (mean 1350 mL, range 500–3900) and required intraoperative blood (mean 4 units, range 2–8) and plasma (mean 6 units, range 2–10) transfusions.

Most patients (16, 57.1 %) had an uneventful recovery. The only HIPEC-related adverse event was a grade 1 renal cisplatin toxicity reversed by medical treatment.

Grade 1/2 complications developed in six (21.4 %), grade 3 in two (7.1 %), and grade 4 in four (14.2 %) patients. Of the four patients with grade 4 complications, two underwent a second operation for fistulas (one colonic and one small bowel) caused by the surgical maneuvers needed to ablate bowel implants, one for postoperative bleeding, and one for an abdominal eventration. Mean postoperative stay was 19.2 days (range 8–71).

**Fig. 1:**
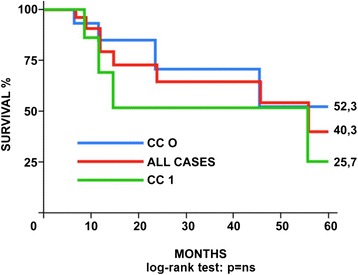
CRS + HIPEC: Kaplan Meier 5-year survival curves according to the completeness of cytoreduction (CC) score

Median survival for the study group was 56 months, and 5-year overall survival reached 40.3 %, with a difference between CC0 and CC1 patients (52.3 vs. 25.7), not reaching statistical significance (Fig. 1). Ten patients are alive disease-free, and eight are alive with disease (Table 1).

## Discussion

### Management of peritoneal metastases from breast cancer

Peritoneal carcinomatosis from breast cancer (BC) is rare but carries high morbidity and mortality [30–32], and no clear guidelines are available regarding the role of CRS with or without HIPEC for those patients [33, 34]. Literature reports are sporadic and only Gusani, one patient in 2008 [1], and Glehen, two patients in 2010 [15], reported PM from BC treated by CRS and HIPEC. Our study provides previously unavailable information on the treatment of women with PM from BC showing that once the correct diagnosis has been established [30], these patients can benefit from treatment and possibly argues against previous reports describing a poor prognosis. In our patients, a median 18 years (range 10–30) elapsed after BC was diagnosed and peritoneal carcinomatosis developed and accords with previous reports describing breast carcinoma as one of the most slowly growing solid tumors given that metastases may appear even decades after the initial diagnosis [34, 35]. Of the five patients treated, four achieved long-term survival, one surviving even for 10 years with good QOL.

Although CRS and HIPEC cannot be proposed as a standard care for patients with PM from primary breast cancer, the survival observed in our small series suggests that in highly selected patients with no extra peritoneal disease and in whom surgery can achieve adequate cytoreduction this combined procedure can offer patients a promising approach for long-term survival.

### Management of peritoneal carcinomatosis from small bowel adenocarcinoma

Management of patients with PM from small bowel adenocarcinoma is unclear with literature reports episodic, even if PM is a frequent manifestation of small bowel carcinoma [36, 37]. Typically, these tumors present after a significant delay in diagnosis for the vagueness of symptoms and imaging difficulty. Prognosis is poor with survival varying from 10 to 40 months. Marchettini and Sugarbaker [38] reported a median survival of 12 months with two of the patients treated with CRS and HIPEC with prolonged survival (57 and 59 months). Chua [39] reported seven cases treated with CRS and HIPEC (mitomycin C and EPIC with 5-FU), with a median disease-free survival of 12 months, and also reported a Kaplan-Meier analysis for a combined group of 19 patients treated with CRS and HIPEC with a median survival of 29 months. Shen et al. [40] reported a median survival of 45 months after treatment with CRS and HIPEC. Another large multi-institutional experience is reported by the French Surgical Association [41], with a median survival for patients treated by CRS and HIPEC of 32 months. In the four patients treated in our Institution, mean survival was 31.2 months, with two patients alive disease-free at 43 and 22 months and two alive with disease at 33 (pulmonary metastases) and at 27 (abdominal recurrence) months. All the series reported show better results when compared to conventional treatments. Moreover, it has to be considered that CRS and HIPEC could represent the only valid surgical option for palliation in obstructed patients, in whom a simple surgical procedure aimed at bowel decompression is often impossible due to small bowel mesentery retraction or for the treatment of associated ascites.

### Management of peritoneal carcinomatosis from serous papillary (type II) uterine carcinoma (UPSC)

Endometrial cancer is still the most common cancer of the female reproductive tract, and its treatment is surgical, alone or in combination with brachy and/or radiotherapy. Survival rates are approximately 90 % at 5 years [42]. When compared to type I tumors, type II endometrial cancers are more likely to present or develop metastatic disease and have a less favorable diagnosis. In the presence of peritoneal metastases, the management becomes more complex and prognosis is poor, with a median survival not reaching 1 year. Bakrin [43] reported five patients with endometrial cancer treated by this combined modality, with a median survival of 19.4 months. Two patients experienced recurrent disease and died, while three patients are alive disease-free at 7, 23, and 39 months after treatment. Glehen [44] in a multi-institutional review of the French Surgical Association of 1290 patients with peritoneal metastases from various primary tumors reported the treatment of 17 patients with uterine adenocarcinoma and epidermoid carcinoma (4 patients), failing to give specific survival data for this specific group of patients. Delotte [42] in 2014 reported CRS and HIPEC in 13 patients with endometrial cancer. Five patients died of the disease and three are alive with disease at 14, 26, and 28 months, while four patients are alive disease-free at 1, 60, 60, and 124 months. In our Institution, we treated eight patients with a diagnosis of type II UPSC with CRS and HIPEC. In four patients, we observed recurrent disease, and two of them died of the disease at 9 and 13 months, while two are alive with disease at 19 and 26 months. Four patients are alive disease-free at 9, 14, 26, and 33 months. Treatment strategies for stage IV endometrial cancer remain controversial. Some reports highlight the histologic characteristics and extent of the disease as the main prognostic determinants, while others favor the effects of a more aggressive surgical cytoreduction. The long-term survival reported in these observational studies, higher when compared to those reported in literature with conventional treatments, seems to justify a more aggressive surgical attitude with the aim to leave the patients without residual visible disease.

### Management of peritoneal carcinomatosis from imatinib-resistant GISTosis

Survival of patients with gastrointestinal stromal tumors (GIST) greatly improved with the clinical use of molecular-targeted therapies [45]. However, the prognosis of imatinib-resistant GIST disseminated to the peritoneum (spontaneously or during surgery) is poor. Accepted conventional treatments including palliative surgery, chemo, and/or radiotherapy are ineffective [46]. As with PM from other gastrointestinal or gynecologic epithelial tumors, a strong rationale favors aggressive locoregional treatment in these patients including peritonectomy procedures combined with HIPEC [47–48] even if its use is controversial due to the rarity of the condition and the few available published reports [49]. The results of our small series of three small bowel imatinib-resistant GIST treated with CRS and HIPEC (two patients alive disease-free at 34 and 108 months, one patient died of disease at 38 months) are in line with similar reports (Table 2) and compare favorably with historical control groups justifying an effort to optimize treatment of the primary or recurrent GISTosis.

**Table 2:** CRS + HIPEC for PC from various primary tumors

Author (year)	Primary tumor	Number	Overall survival			
			Median (Months)	1 year (%)	3 years (%)	5 years (%)
Jacks (2005) [37]	Small bowel	6	30	-	-	-
Gusani (2008) [1]	Unknown	2	26.2	-	49	-
	Breast	1				
	GIST	6				
	Gallbladder	1				
	Liver	1				
	Adrenal	1				
	Esophagus	1				
Shen (2009) [40]	Unknown	2	22.2	66	40	27
	Pancreas	5				
	GIST	11				
	Sarcoma	10				
	Gallbladder	3				
	Adrenal	1				
	Small bowel	6				
	Urachus	5				
Chua (2009) [3]	Small bowel	7	25	57	20	-
Kerscher (2010) [50]	Small bowel	3		-	-	-
Glehen (2010) [15]	Unknown	8	34	77	49	37
	Breast	2				
	GIST	3				
	Sarcoma	28				
	Liver	2				
	Adrenal	3				
	Urachus	4				
	Small bowel	45				
	Esophagus	1				
	Kidney	2				
Turrini (2012) [52]	Pancreas	1	-	-	-	-
Randle (2013) [48]	Sarcoma	10	21	-	-	43

Review of the literature

### Management of peritoneal carcinomatosis from other unconventional miscellaneous tumors

The optimal management of patients with rare and unusual primary tumors metastatic to the abdominal cavity is a matter of intense debate. Systemic chemotherapy for PM improved but remains limited because of poor diffusion of the drugs into the peritoneum. This is why many authors [1, 34, 35, 50–55] reported small observational series of patients with PM from various unconventional tumors treated by CRS and HIPEC (Table 2). This combined treatment modality has been used in peritoneal metastases from pancreatic, abdominal sarcomas, gallbladder, liver, cholangiocarcinoma, adrenal, urachal, esophageal, and kidney tumors. In a multi-institutional review of the French Surgical Association on 1290 cases of PM from various primary tumors treated with CRS and HIPEC [41], the unconventional indications were 29. Mortality was 4.1 % with a rate of major (grade 3 and 4) complications of 33 %, similar to those reported after other major surgical procedures. Obviously, the numbers are too small to draw any conclusion on survival figures for each specific primary tumor, but an overall median survival of 34 months, with a 5-year disease-free survival of 22 %, compares favorably with survival figures reported in literature of palliative treatments for the same tumors.

## Conclusions

We can conclude that CRS and HIPEC should not be excluded “a priori” for the treatment of peritoneal metastases from rare or unusual (“unconventional”) primary tumors. This combined multimodality therapeutic approach, performed in selected patients in an experienced peritoneal surface malignancy center, is safe and has shown to provide not only a better palliation but also a survival benefit over conventional palliative treatments.
